# Epidemiological insights and genetic diversity of the Duffy binding protein of *Plasmodium vivax* in Duffy-negative Cameroonians

**DOI:** 10.1371/journal.pntd.0014404

**Published:** 2026-06-04

**Authors:** Cheikh Cambel Dieng, Rene Teh Ning, Canelle Kipayko, Regan Elizabeth Schroeder, Nontokozo Mdluli-Berndt, Bate Ayukenchengamba, Zidedine Nematchoua Weyou, Tabi Doris Sona, Ambendekson Elizabeth Reward, Guofa Zhou, Irene Sumbele Ngole Ule, Helen Kuokuo Kimbi, Eugenia Lo

**Affiliations:** 1 Department of Microbiology and Immunology, College of Medicine, Drexel University, Philadelphia, Pennsylvania, United States of America; 2 Department of Animal Biology and Conservation (ABC), Faculty of Science, University of Buea, Buea, Cameroon; 3 Department of Population Health and Disease Prevention, School of Public Health, University of California at Irvine, Irvine, California, United States of America; 4 Department of Biomedical Sciences, University of Bamenda, Bamenda, Bambili, Cameroon; Swiss Tropical and Public Health Institute: Schweizerisches Tropen- und Public Health-Institut, SWITZERLAND

## Abstract

**Background:**

Malaria remains a major public health concern in sub-Saharan Africa. *Plasmodium vivax* (*P. vivax*), historically considered rare due to the predominance of Duffy-negative individuals, is increasingly reported in Central and West Africa. The ability of *P. vivax* to infect Duffy-negative populations challenges long-standing assumptions regarding parasite invasion biology and highlight surveillance gaps across Africa.

**Methodology/Principal findings:**

This study investigated *P. vivax* prevalence and genetic diversity across three ecological zones in Cameroon. A total of 1,373 samples were screened by microscopy, rapid diagnostic tests (RDTs), and qPCR; and all participants were genotyped for Duffy antigen status. *PvDBP1* region II was successfully sequenced from 75 *P. vivax* isolates. *P. vivax* prevalence was 10.8% among hospital patients (86/793) and 5.5% in community participants (32/580), and all confirmed infections occurred in Duffy-negative individuals. Hospital infections exhibited significantly higher parasitemia than asymptomatic cases. PvLDH-based RDTs failed to detect over 85% of qPCR-confirmed infections. Genetic analysis of *PvDBP1* identified eight nonsynonymous mutations, with I379L (74.1%) and E225K (61.3%) as the most common variants, suggesting possible adaptive evolution. Phylogenetic analysis clustered Cameroonian *P. viv*axisolates with those from Botswana, distinct from East African and Asian lineages, indicating regional adaptation and potential gene flow within Central-Southern Africa.

**Conclusion/Significance:**

This study provides the first integrated epidemiological and *PvDBP1* genetic characterization of *P. vivax* infections in Duffy-negative Central Africans, revealing widespread subclinical infections and poor diagnostic performance of current PvLDH-based RDTs. The observed genetic signatures of adaptation highlight the urgent need to prioritize *P. vivax* within national malaria programs and investigate alternative invasion pathways beyond *PvDBP1* to guide improved diagnostic and vaccine strategies.

## Author summary

*Plasmodium vivax* (*P. vivax*) malaria has traditionally been considered rare in sub-Saharan Africa because most people lack the Duffy blood-group antigen, which was thought to be required for the parasite to invade red blood cells. However, recent reports show that *P. vivax* can indeed infect Duffy-negative individuals, suggesting that its burden in Africa has been underestimated. In this study, we investigated the prevalence of *P. vivax* infection in three regions of Cameroon and analyzed the genetic features of circulating parasites. Using sensitive molecular tests, we detected *P. vivax* in both febrile hospital patients and asymptomatic community members, all of whom were Duffy-negatives. Standard rapid diagnostic tests and microscopy missed the great majority of these infections, even at high parasite loads. Genetic analysis of the parasite’s Duffy Binding Protein (*PvDBP1*) revealed frequent mutations unique to the Central African isolates, suggesting the parasite may be adapting to local Duffy-negative populations. Our results show that *P. vivax* is silently circulating in Cameroon and that its burden is underestimated by current surveillance approaches. Addressing *P. vivax* malaria in Africa will require improved diagnostic strategies and deeper investigation into the parasite’s evolving invasion mechanisms.

## Introduction

Malaria remains a global health challenge in sub-Saharan Africa. For decades, *Plasmodium vivax* (*P. vivax)* was thought to be rare in African populations because most individuals are Duffy-negative, lacking erythrocyte surface expression of the Duffy antigen receptor for chemokines (DARC). *P. vivax* invasion relies on the interaction between its Duffy Binding Protein 1 (*PvDBP1*) and DARC during the asexual blood stage of infection. This paradigm led to the longstanding assumption that Duffy negativity confers protection against *P. vivax*. However, an increasing number of molecular studies have identified *P. vivax* cases in Duffy-negative individuals across Africa, including Equatorial Guinea and Angola [1], the Democratic Republic of Congo [2], Brazil [3], Ethiopia [4,5], Madagascar [6], Kenya [7], Mauritania [8], Cameroon [9,10], Mali [11], and Benin [12], demonstrating that Duffy-negativity does not confer complete resistance to infection. The endemic range of *P. vivax* has extended beyond the Horn of Africa and penetrated Duffy-negative regions (>95%). A point mutation (c.1–67T > C; rs2814778) in the GATA-1 transcription factor binding site of the Duffy antigen/receptor for chemokines (DARC) gene promoter alters erythroid expression, reducing Duffy antigen expression on the surface of red blood cells [13,14].

In Cameroon, the highest malaria prevalence occurs in the western and northern highland regions, which exhibit different transmission dynamics compared to the lowlands in the eastern region [15]. Malaria prevalence also varies markedly by season, ranging from 30% in the dry season from November to February to 65% during the rainy season from March to October. The western part of Cameroon experiences heavy seasonal rainfall, which drives malaria transmission. While *P. falciparum* (*P. faciparum*) remains the dominant parasite species, *P. vivax* cases have been reported among travelers who acquired infections in Cameroon and were diagnosed after returning to their home countries [16,17]. *P. vivax*-like asymptomatic infections have also been detected by microscopy in schoolchildren in the Mount Cameroon area. In addition, two recent studies reported an infection rate of 5.6% [18] and 35% [9] of *P. vivax* in febrile Duffy-negative individuals in Dschang, northwestern Cameroon by PCR. However, these studies were case reports and did not assess the broader epidemiological or genetic characteristics of *P. vivax* in the region. Further, the extent of genetic polymorphisms of *PvDBP1,* particularly region II of the DARC-binding domain [19], has not been characterized in Central African *P. vivax* populations. Understanding parasite diversity and evolution in these settings is essential for identifying potential adaptations that support Duffy-independent erythrocyte invasion, which would have major implications for surveillance, diagnostics, and vaccine design in Africa.

Environmental factors such as temperature, rainfall, and elevation can shape *P. vivax* transmission dynamics through their effects on mosquito vectors and parasite development. Across Cameroon’s diverse ecological landscapes, these conditions may influence regional patterns of *P. vivax* transmission [20]. In this study, we assessed *P. vivax* prevalence among the northwestern, southwestern, and eastern regions of Cameroon in both community and hospital settings. We further examined *PvDBP1* genetic polymorphisms from these regions and compared them to isolates from East Africa and beyond, to evaluate genetic diversity and infer evolutionary relationships. Understanding the epidemiological and genetic features of *P. vivax*, particularly in Duffy-negative populations, is essential for informing malaria surveillance and control strategies in Africa.

## Methods

### Ethical statement

The study protocol was approved by the Institutional Review Boards of the Universities of Buea and Bamenda (Ref No. 2024/2110-06/UB/SG/IRB/FHS). Written informed consent was obtained from all participants or, in the case of minors, from their parent or legal guardian. All methods were carried out in accordance with relevant guidelines and regulations.

### Study sites and sample collection

A cross-sectional study was conducted across three study sites in Cameroon including Bamenda, Buea, and Bertoua. Site selection was based on variation in climate and landscape alongside prior reports of *P. vivax* in nearby areas. Bamenda lies in a highland region (1201–1400 m). Buea, at 530 meters elevation, is a multi-ethnic semi-urban area with year-round malaria transmission [10,21]. Bertoua, in the Eastern region, has a wet equatorial climate and is known for timber and mining industries [20]. A total of 1,373 samples from Buea (*n* = 408, of which 398 passed DNA-quality control), Bamenda (*n* = 498), and Bertoua (*n* = 467) were collected between April and August 2023. Among all samples, 793 were from febrile patients at hospitals (3 in Buea, 1 in Bamenda) and 580 from households in surrounding communities (Buea and Bertoua); 555 were males and 818 were females. Hospitals were selected based on accessibility and patient volume and community households were randomly selected. The relatively low number of children in the Buea community reflects daytime household availability and caregiver consent patterns. For each individual, 30–50 μL of finger-pricked blood was spotted onto Whatman 3MM filter paper, air-dried, and stored until DNA extraction. Thick and thin blood smears were also prepared for microscopic screening of *Plasmodium* species.

### *Plasmodium* screening, Duffy genotyping, and *PvDBP1* sequencing

*Plasmodium* species were initially determined using rapid diagnostic tests (RDTs; Bioline Malaria Ag P.f/Pv manufactured by Abbott Inc) specific to *P. falciparum* (PfHRP2) and *P. vivax* (PvLDH), as well as microscopy. Parasitic DNA was extracted from dried blood spots using a modified Chelex-saponin method [4,22]. Quantitative PCR (qPCR) targeting the 18S rRNA gene was used to identify mono-*P. vivax* and mixed *P. vivax-P. falciparum* infections [23]. Parasitemia was measured via SYBR Green detection method, using *P. vivax*-specific primers (S1 Table) [4]. Each run included positive controls from *P. vivax* Pakchong (MRA-342G) and Nicaragua (MRA-340G) isolates, along with negative controls. A standard curve was generated based on a ten-fold serial dilution of a *P. vivax* control plasmid to assess amplification efficiency (E). Specificity of the amplification was verified by melting curve analysis. For each sample, the mean cycle threshold (Ct) value and standard error were calculated from three independent runs. Parasitemia was determined using the equation: 2^E × (40 - Ct__sample_).

In addition, the gene encoding the Duffy Binding Protein (*PvDBP1*) located on chromosome 6 (PlasmoDB: PVP01_0623800) was analyzed. For each sample, *PvDBP1* region II was amplified using published primers (forward: 5′-GATATTGATCATAAGAAAACGATCTCTAGT-3′; reverse: 5′-TGTCACAACTTCCTGAGTATTTTTTTTAGCCTC-3′) [24], designed based on the *P. vivax* reference sequence PVX_110810 from the Sal I strain (NC_009911.1). The amplification was performed following the published protocol [24]. Sequencing of the *PvDBP1* gene region II was performed by Sanger sequencing. Chromatograms were visually inspected, bases with Phred < 30 were trimmed, and consensus sequences were generated in Geneious v2025.0.2. For host Duffy genotyping, a TaqMan qPCR assay was used to examine the point mutation (c.1–67T > C; rs2814778) of the *DARC* gene [25]. A no-template control was used in each assay. Genotypes were determined by the allelic discrimination plot. In addition, a 1,100-bp fragment of the *DARC* gene was further amplified for a subset of samples using published primers for Sanger sequencing to confirm Duffy genotypes [6].

### Data analysis

All statistical analyses were conducted within the JupyterLab Desktop (version 4.2.5-1) environment. Analyses were restricted to *a priori* defined variables of epidemiological relevance, including age group, sex, and sampling origin (hospital vs. community). Associations between infection type (mono-*P. vivax*, mono-*P. falciparum*, and mixed infections) and explanatory variables were assessed using univariate logistic regression models. For categorical variables with more than two levels (e.g., age group), overall associations were evaluated using likelihood ratio tests comparing models with and without the variable of interest. The overall *p*-values were reported in addition to odds ratios (ORs) and 95% confidence intervals (CIs) to estimate effect sizes. No formal correction for multiple comparisons was applied (e.g., Bonferroni), as analyses were limited to a small number of pre-specified, hypothesis-driven tests.

Reported symptoms (S4 Table) were not included in inferential statistical analyses due to inconsistent data collection across sites and potential confounding from non-malaria febrile illnesses. While samples were collected within health facilities and communities, suggesting a degree of natural clustering, all analyses assume independence of observations. The potential for intra-cluster correlation was not explicitly modeled and is considered as a potential limitation.

*PvDBP1* sequences were aligned to the P01 (PlasmoDB: PVP01_06230800) reference strain using MUSCLE in Geneious vX.1*.* Comparative sequences from Thailand (*n* = 10) [26] and China (*n* = 18) (Asia), Brazil (*n* = 15) [27] (South America), Sudan (*n* = 60) [28] and Ethiopia (*n* = 78) [29] (East Africa), and Botswana (*n* = 12) [30] (Southern Africa) that represent geographically and epidemiologically diverse regions were included. For comparative phylogenetic analysis, *PvDBP1* region II sequences were obtained from two sources: (i) Sanger-sequenced *PvDBP1* region II amplicons generated in this study; and (ii) *PvDBP1* region II regions extracted from publicly available whole-genome sequencing datasets (accession numbers listed in S2 Table). Only *PvDBP1* region II was used for tree inference; no whole-genome phylogenies were constructed. Reads were quality-filtered using Trimmomatic v0.39, aligned to the *Pv*P01 reference genome using BWA-MEM, and variants were called using GATK HaplotypeCaller following best practices. Consensus sequences for the *PvDBP1* region II (codons 291–460) were extracted using bcftools and manually checked for completeness. Only sequences with <5% ambiguous bases were retained. Phylogenetic analyses were conducted using the Maximum Likelihood (ML) approach implemented in RAxML v8.2.12. The alignment included *PvDBP1* region II sequences generated in this study and publicly available global isolates (Thailand, India, Ethiopia, Sudan, Brazil, Botswana, and Uganda; see S2 Table for accession numbers and sample counts). The ML tree was inferred under the General Time Reversible (GTR) model with a gamma-distributed rate variation among sites and 1,000 bootstrap replicates to assess node support. The final tree was visualized in FigTree v1.4.5, with clades colored by geographic origin. All *PvDBP1* region II sequences of the Cameroonian *Pv* samples obtained in this study were deposited in GenBank (accession number PX699213 - PX699280).

## Results

### Prevalence of *P. vivax* infections among Duffy-negatives

**Fig 1:**
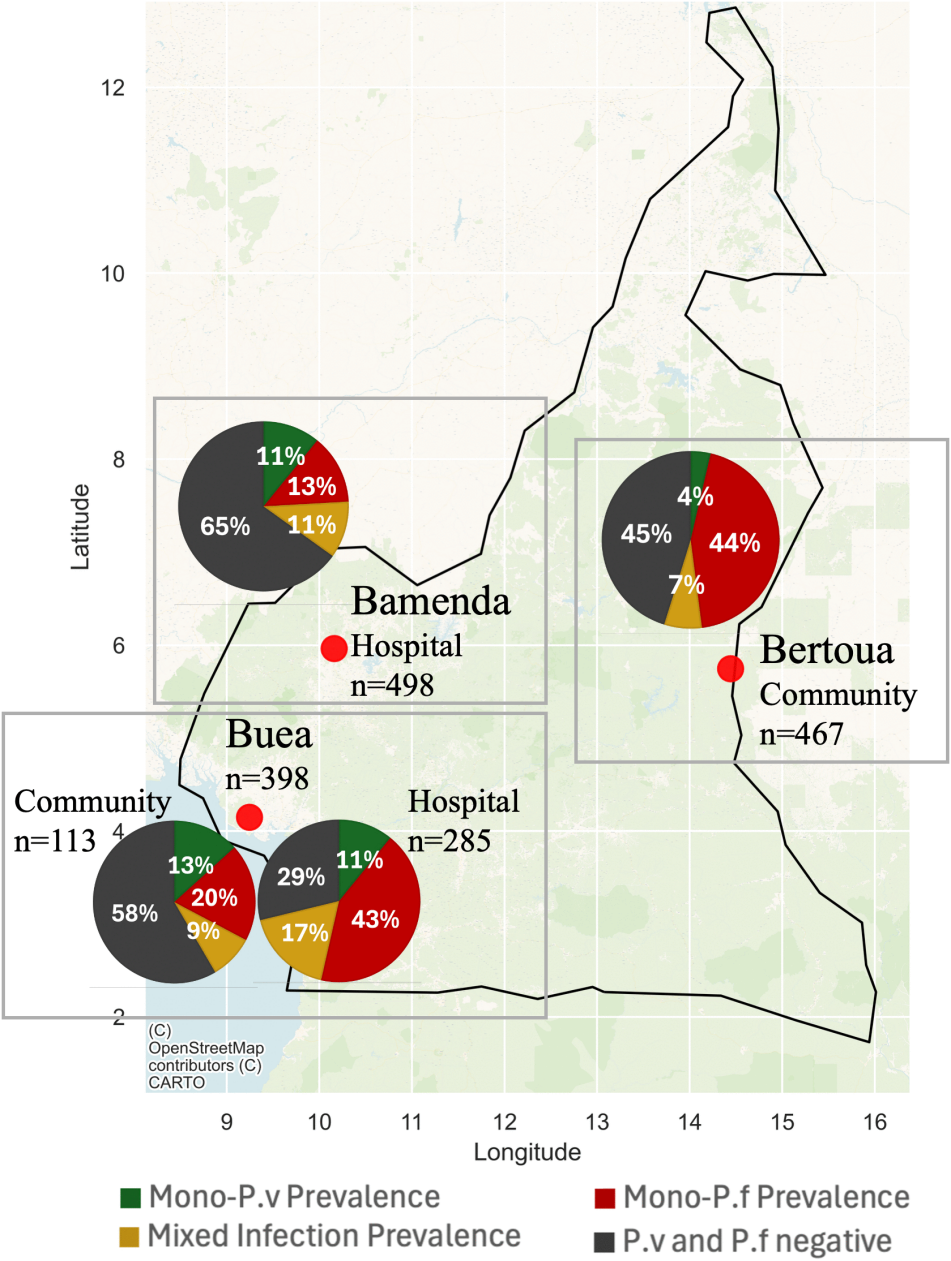
Study sites across Cameroon (Bamenda, Buea, and Bertoua) by hospital and community-based collection and infection type including mono-*P. vvivax*, mono-*P.* *Falciparum*, mixed *P. vivax* and *P. falciparum*, and negative samples. The prevalence of mono-*P. vivax* and mixed *P. vivax*-*P. falciparum* infections showed variation across the study sites. Map was created in JupyterLab Desktop version 4.2.5-1. Map base layer link: https://github.com/datasets/geo-countries.

**Fig 2:**
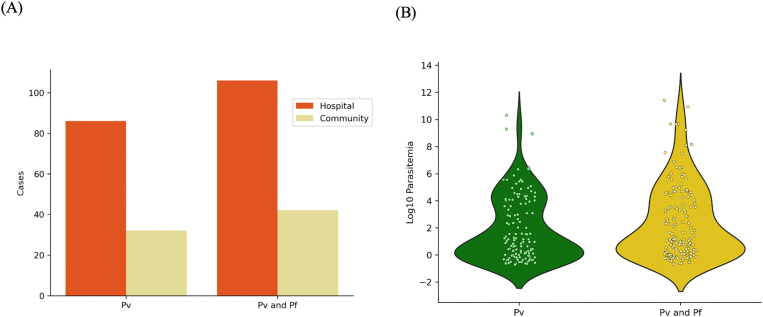
Prevalence of *Plasmodium vivax (P. vivax)* and *Plasmodium falciparum (P. falciparum)* infections across hospital and community settings and Parasitemia levels in mono and mixed infections. **(A)** Panel shows the number of cases for *P. vivax* and mixed *P. vivax* and *P. falciparum* infections across hospital (orange bars) and community (olive green bars) settings. Among the hospital samples, 10% (86) were positive for *P. vivax* while 52.3% (409) samples showed mixed infections, with both *P. vivax* and *P. falciparum* present. In community samples, 5.5% (32) were positive for *P. vivax* and 47.8% (277) samples showed mixed infections. **B)** *P. vivax* parasite load between mono-*P. vivax* and mixed *P. vivax* and *P. falciparum* infections (*p* = 0.6542 by Mann–Whitney U test).

The prevalence of mono-*P. vivax* and mixed *P. vivax-P. falciaprum* infections varied substantially across study sites (Fig 1). Among 793 hospital samples, mono-*P. vivax* prevalence was 10.8% (*n* = 86), mono-*Pf* was 24.2% (*n* = 192), and mixed *P. vivax-P. falciparum* infections were 13.4% (*n* = 106; Fig 2A, Table 1). Mono-*P. vivax* positivity rates were similar between Bamenda and Bertoua, whereas Buea has significantly higher mono-*P. falciparum* and mixed infections compared to Bamenda (χ² = 119.97, df = 3, *p* < 0.001). However, the prevalence of mixed infections did not vary significantly among sites (χ² = 0.80, df = 2, *p* = 0.67). All *P. vivax* infections were Duffy-negative (CC) per TaqMan genotyping.

**Table 1:** Prevalence of mono-*Plasmodium vivax*, mono-*Plasmodium falciparum*, and mixed infections across hospital and community samples in Bamenda, Bertoua, and Buea. The infection prevalence of mono-*Pv*, mono-*Pf*, and mixed *Pv* and *Pf* infections were indicated across three sites in Cameroon (Bamenda, Bertoua, and Buea). Data are stratified based on collection setting (hospital vs community) and geographic location. For each site, the percentage distribution of infections and the total number of cases are provided.

Site	Total (N)	Mono-*Pv*	Mono-*Pf*	Pv + Pf
**Hospital**		**Positivity rate (%, n)**		
Bamenda	498	10.8 (54)	13.3 (66)	10.8 (54)
Buea	295	17.7 (32)	42.7 (126)	17.6 (52)
Total (%)	793	10.8 (86)	24.2 (192)	13.4 (106)
**Community**		**Prevalence rate (%, n)**		
Bertoua (>15)	140	2.9 (4)	36.4 (51)	8.6 (12)
Buea (>15)	112	12.5 (14)	19.6 (22)	8.9 (10)
**Total (%)**	252	7.1 (18)	29.0 (73)	8.8 (22)

Among 580 community samples, mono-*Pv* prevalence was 5.5% (n = 32), mono-*Pf* was 39.5% (*n* = 229), and mixed *P. vivax-P. falciaprum* infections were 7.2% (*n* = 42) (S3 Table). The infection rates were assessed only for individuals aged >15 due to limited samples of the younger age groups (see S3 Table for data stratified by age). Buea showed significantly higher mono-*P. vivax* infections than Bertoua (χ² = 8.13, df = 1, *p* = 0.004). The highest prevalence of mixed infections was observed in Bamenda (36.54%) and Bertoua (34.26%), followed by Buea (29.20%) with no significant differences (χ² = 0.80, df = 2, *p* = 0.67).

### Association of demographic factors with infection type

No significant difference was observed in *P. vivax* infection between males and females (OR = 0.90, 95% CI [0.61–1.33], *p* = 0.60; Table 2). Similarly, the odds of mixed infection did not differ by sex (OR = 0.91, 95% CI [0.64–1.30], *p* = 0.60), and no association was observed between sex and *P. falciparum* infection (*p* = 0.84). On the other hand, age group was significantly associated with infection type (*p* < 0.01). Compared to children under 5 years old, individuals aged 5–15 had similar odds of *P. vivax* infection (OR = 0.98, 95% CI [0.51–1.88]), but significantly higher odds of *P. falciparum* infection (OR = 1.64, 95% CI [1.20–2.25]; Table 2). In contrast, individuals older than 15 years old had significantly higher odds of *P. vivax* infection (OR = 2.08, 95% CI [1.29–3.77]) and lower odds of *P. falciparum* infection (OR = 0.67, 95% CI [0.51–0.88]) than other age groups. There was no evidence of a significant association between age group and mixed infection (*p* = 0.79). Sampling origin was strongly associated with infection type (*p* < 0.001). Mono-*P. vivax* and mixed infections were more common in hospital-based participants (72.9% and 71.6%) than those from the community (27.1% and 28.4%). Compared to hospital-based samples (Table 2), community samples had lower odds of *P. vivax* (OR = 0.48, 95% CI [0.32–0.73]) and mixed infection (OR = 0.51, 95% CI [0.35–0.74]), but higher odds of *P. falciparum* infection (OR = 2.04, 95% CI [1.62–2.58]). Although infection prevalence varied by sexes and age groups, no significant differences were observed in parasitemia between mono- and mixed infections (Fig 2B), sexes (S1 Fig), or age groups (S2 Fig).

**Table 2:** Demographic and Recruitment Setting (Hospital vs Community) Associated Risk Factors for *P. vivax*, *P. falciparum*, and mixed Infections. The distribution of malaria infections across various demographic and geographic categories were presented. *, **, and *** represent significant at 0.05, 0.01, and 0.001 levels, respectively.

Variables	N Samples	Mono-P*v* OR [95% CI]	Mono-*Pf* OR [95% CI]	*Pv* + *Pf* OR [95% CI]	Overall p-value
Sex					**0.84**
Female	818	1	1	1	
Male	555	0.90 [0.61, 1.33]	1.15 [0.91, 1.45]	0.91 [0.64, 1.30]	
Age					<0.01**
< 5	396	1	1	1	
5–15	282	0.98 [0.51, 1.88]	1.64 [1.20, 2.25]	0.86 [0.52, 1.41]	
> 15	695	2.08 [1.29, 3.77]	0.67 [0.51, 0.88]	0.94 [0.64, 1.40]	
Origin					<0.001***
Hospital	793	1	1	1	
Community	580	0.48 [0.32, 0.73]	2.04 [1.62, 2.58]	0.51 [0.35, 0.74]	

### Substantial variations in detection limit

**Fig 3:**
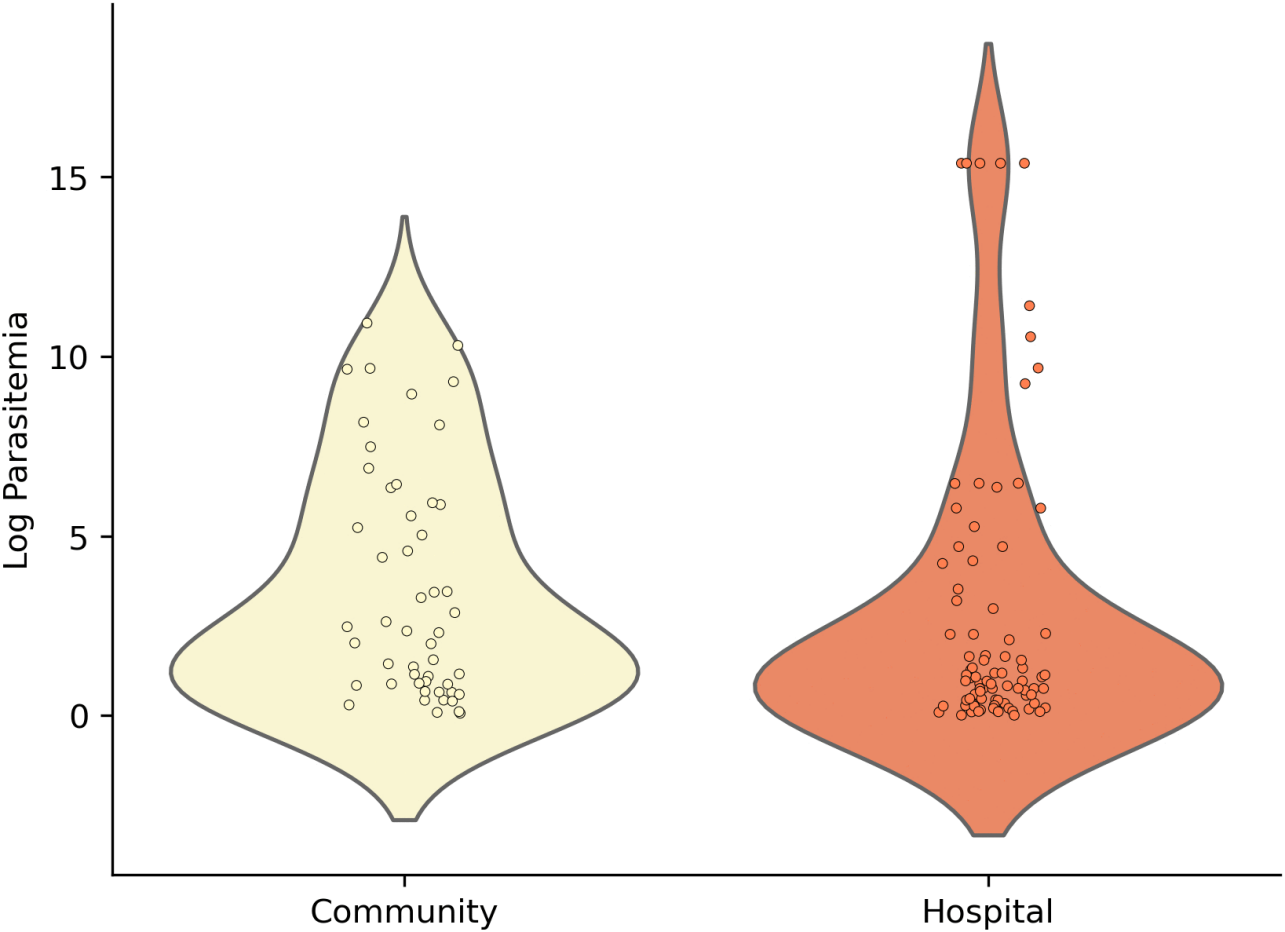
Analysis of parasitemia levels by sample origin in *P. vivax* infections. Parasitemia levels between hospital and community samples, showing significantly higher parasitemia in hospital samples.

Of 119 *P. vivax*-positive samples by 18S-qPCR, 112 (94%) were confirmed by *PvDBP*1-based PCR. However, all qPCR-positive samples were microscopy-negative, and only 15 (12.6%) were RDT-positive (Table 3). These 15 RDT-positive samples showed relatively low Ct values (i.e., high parasite DNA copies) by qPCR (Table 3). These findings highlighted the limited sensitivity of conventional diagnostics in detecting *P. vivax* infections, particularly in Duffy-negative individuals. While hospital samples had significantly higher average parasitemia than community samples (*p* < 0.001; Fig 3), several community-based (asymptomatic at the time of collection) infections were shown with relatively high parasitemia. Specifically, 13% of community samples had parasitemia >1,000 parasites/μL, compared to 21% of hospital cases showing such level of parasitemia. Overall, most *P. vivax* infections, particularly those with higher Ct values (indicative of lower parasite densities), were undetectable by microscopy and RDT. All qPCR-positive *P. vivax* samples were from Duffy-negative individuals.

**Table 3:** Detection of *P. vivax* Infections using 18S-qPCR, *PvDBP1*-targeted PCR (DBP-PCR), microscopy, and rapid diagnostic tests (RDTs) in hospital-based (*n=* 793) and community-based (*n=* 580) samples. Most *P. vivax* infections, particularly those with higher Ct values (indicative of lower parasite densities), were undetectable by microscopy and RDT. All qPCR-positive *P. vivax* samples were from Duffy-negative individuals.

Ct range by qPCR	18S-based qPCR	DBP-based PCR+	Microscopic+	RDT+
Hospital-based samples (total N = 793)				
< 20	0	0	0	0
20– < 25	5	5	0	5
25– < 30	17	17	0	7
30– < 35	20	18	0	0
> 35– < 38	45	43	0	0
Community-based samples (total N = 580)				
< 20	3	3	0	3
20– < 25	3	2	0	0
25– < 30	1	1	0	0
30– < 35	4	4	0	0
> 35– < 38	21	19	0	0
**Total**	**119**	**112**	**0**	**15**

### Genetic polymorphisms of *PvDBP1* and phylogenic relatedness

**Fig 4:**
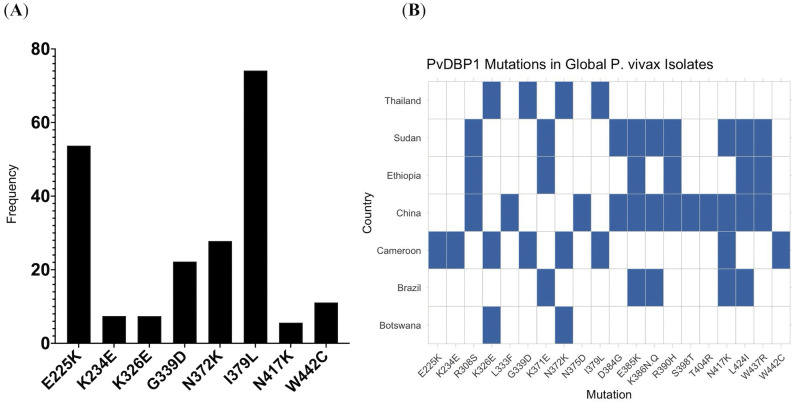
Distribution of DBP gene mutations in *P. vivax* isolates from Cameroon and global comparisons. **(A)** The bar chart illustrates the frequency of *Plasmodium vivax* Duffy Binding Protein (DBP) gene mutations in isolates from Cameroon. The mutations E225K, K234E, K326E, G339D, N372K, I379L, N417K, and W442C were observed with varying prevalence, with I379L showing the highest frequency. **(B)** Comparison of *PvDBP1* Region II mutations observed in *P. vivax* isolates from Cameroon with those reported in geographically and epidemiologically diverse regions, including Thailand and China (Asia), Brazil (South America), Sudan and Ethiopia (East Africa), and Botswana (Southern Africa). Cameroon shared four mutations (K326E, G339D, N372K, and I379L) with Thailand, though the frequencies of I379L and G339D were lower in Thai isolates. K326E and N372K were also found in Botswana. Brazilian and Sudanese/Ethiopian isolates exhibited a broader mutation profile, including several variants not found in Cameroon. Chinese isolates displayed unique mutations such as L333F, S398T, and T404R. The Cameroonian *P. vivax* exhibited three mutations, E225K, K234E, and W442C, not found in other geographical regions.

Eight mutations were identified in the *PvDBP1* region II (amino acid 291–460) among 75 *P. vivax* isolates from Cameroon. Two of the mutations occurred at high frequency in the Duffy-negative populations, including I379L observed in 74.1% and E225K in 53.7% of the samples (Fig 4A). Other less frequent mutations included N372K (27.8%), G339D (22.2%), W442C (11.1%), K234E (47.1%), K326E (7.4%), and N417K (5.6%). Variants K326E, G339D, N372K, and I379L were shared with the Thai isolates, though I379L (30%) and G339D (19%) were observed at considerably lower frequencies in Thailand (Fig 4B). Only K326E and I379L were shared between the Cameroonian and Botswana isolates. The Chinese *P.vivax* were the most polymorphic with 12 variants detected in the binding region, four of which (L333F, N375D, S398T, and T404R) were unique and not observed elsewhere. The remaining nine variants (L333F, K326E, G339D, N372K, I379L, N417K, S398T, T404R, and W442C) were overlapped with the Sudanese, Ethiopian, and Brazilian isolates. However, none of these variants were detected in the Cameroonian or Botswana *P. vivax*. The Cameroonian *P. vivax* shared N417K with the Brazilian, Sudanese, and Chinese isolates (Fig 4B).

**Fig 5:**
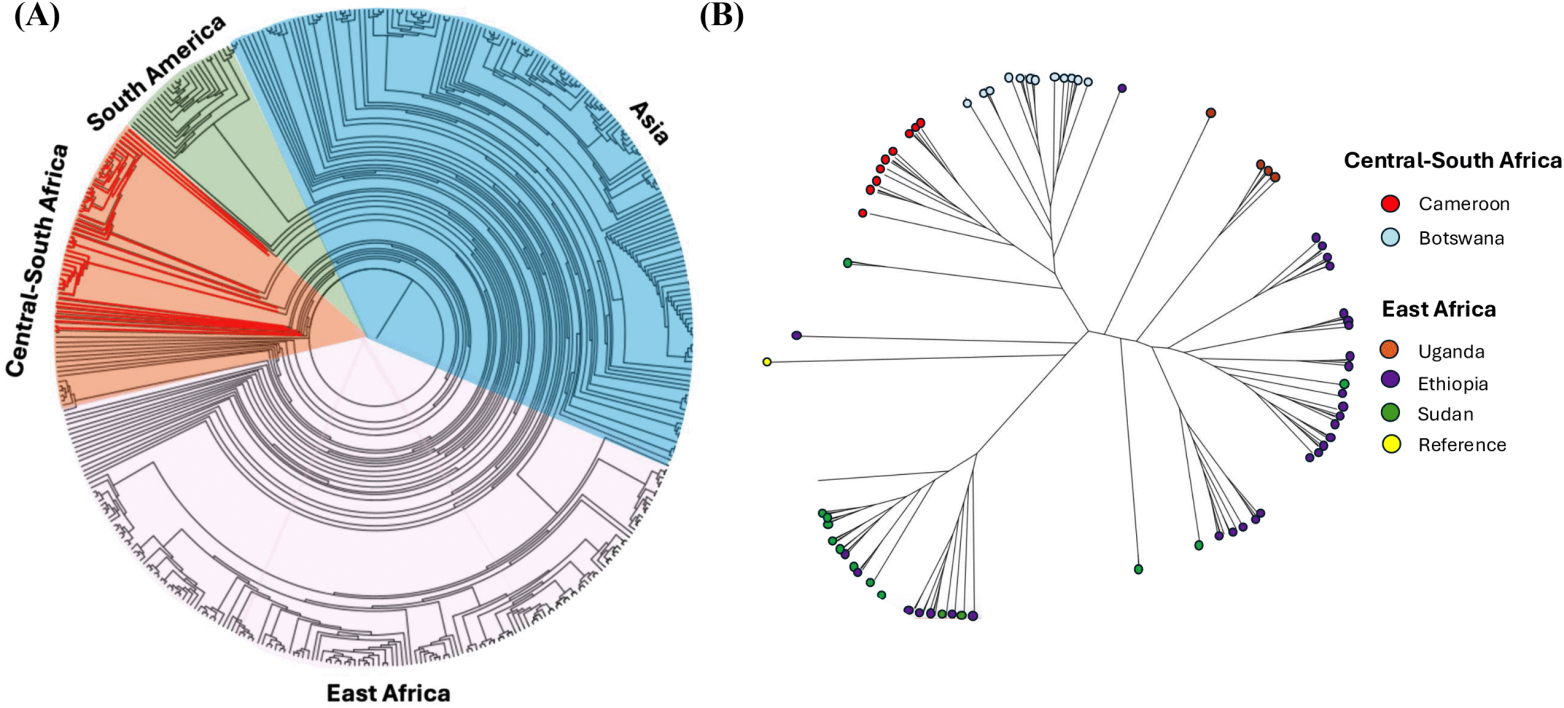
Global and regional phylogenetic relationships of *Plasmodium vivax* isolates. This figure illustrates the phylogenetic relationships of *Plasmodium vivax* isolates from different geographical regions constructed from *PvDBP1* region II sequence data using 1,000 bootstrap replicates. **No WGS phylogeny was constructed**: **(A)** Displays a circular phylogenetic tree that segregates *P. vivax* populations into major global regions, including Central-South Africa, South America, Asia, and East Africa; **(B)** Presents a radial phylogenetic tree, with color-coded branches corresponding to isolates from Cameroon (red), Botswana (light blue), Uganda (orange), Ethiopia (purple), Sudan (green), and reference genomes (yellow). Both trees demonstrate the genetic diversity and evolutionary divergence of *P. vivax* across different geographical populations, with substantial clustering observed among isolates from specific regions. The phylogenetic structure highlights potential gene flow and regional adaptation patterns, particularly in East African populations. These insights are crucial for understanding the evolutionary history and transmission dynamics of *P. vivax* on both global and regional scales.

Phylogenetic analysis revealed distinct clustering of *P. vivax* isolates by geographic region (Fig 5A), with East African and Asian *P. vivax* showing high genetic relatedness, and South American *P. vivax* forming a separate clade distinct from the other regions. Within Africa (Fig 5B), most of the isolates from Cameroon (red) form a distinct cluster, sister to those from Botswana (light blue). Two other main clades were observed—one contained most of the Ethiopian (purple) and Ugandan (orange) isolates, and another contained the Ethiopian and Sudanese (green) isolates—indicating the genetic diversity among the African *P. vivax*.

## Discussion

Molecular assays targeting *P. vivax*-specific 18S rRNA and *PvDBP1* revealed a substantial number of *P. vivax* infections across the study regions in Cameroon. These findings contribute to a growing body of evidence that Duffy-negative individuals are not fully protected against *P. vivax* and that the parasites may have well-adapted and circulating more widely in Central Africa than previously recognized [9,25,31]. Previous reports of *P. vivax* infections in Cameroon [9] and in neighboring countries with predominantly Duffy-negative populations, including the Democratic Republic of the Congo [2,32], further support the emergence of a broader regional *P. vivax* transmission network in central Africa. Our findings also align with a growing body of evidence documenting *P. vivax* infections in predominantly Duffy-negative populations across Sub-Saharan Africa. Large-scale modeling by Brazeau et al. (2021) identified non-zero *P. vivax* prevalence across Central and West African regions, including Cameroon, even though routine diagnostics rarely detect these infections [2]. Similarly, Mitchell et al. (2020) and subsequent studies from Equatorial Guinee and Angola [1], the Democratic Republic of Congo [2], Brazil [3], Ethiopia [4,5], Madagascar [6], Kenya [7], Mauritania [8], Cameroon [9,10], Mali [11], and Benin [12] have shown that low-to-moderate levels of *P. vivax* transmission can occur despite high frequencies of Duffy negativity [32]. The prevalence estimates from our hospitals (10.8%) and communities (5.5%) fall within the range of PCR-based findings reported across the region, reinforcing the conclusion that *P. vivax* circulates more widely in Central Africa than is captured by microscopy or *P. falciparum-*focused surveillance systems. In this context, the clustering of Cameroonian and Botswana isolates apart from East African populations in our *PvDBP1* region II phylogeny may reflect distinct regional epidemiological histories and heterogeneous transmission patterns across the African continent.

Geographic and environmental factors strongly influence *P. vivax* transmission dynamics [33]. Instead of uniform transmission across its range, *P. vivax* displays substantial spatial heterogeneity, with focal hotspots shaped by local ecology, vector abundance, and human movement. Our data from Bamenda, Buea, and Bertoua mirror this pattern and align with recent reports of heterogeneous *P. vivax* circulation in other Duffy-negative African settings [2,32,34]. High rainfall and flooding in Buea and Bamenda may enhance transmission compared to Bertoua [35,36]. Recent studies suggest that malaria is expanding its endemic range to higher altitudes, including regions such as the Ethiopian highlands [31]. In Ethiopia, *P. vivax* has expanded into highland areas previously considered malaria-free, with mono-*P. vivax* infection rates of ~8%, similar to our findings in this study. Our cross-sectional data do not allow prediction of future trends in highland malaria. However, the modest differences we observed between Bamenda (highland) and Bertoua (lowland) underscore that *P. vivax* transmission in Cameroon is highly heterogeneous and shaped by local ecological and demographic conditions. Our findings highlight the need for longitudinal, site-specific surveillance to detect how *P. vivax* may establish or persist in diverse ecological zones of Central Africa. Socio-political unrest and large-scale migration in the English-speaking regions of Cameroon since 2016 may have contributed to the spread of *P. vivax* to more parts of Cameroon or beyond [37]. This discrepancy may reflect local epidemiological or sampling differences and warrants further investigation [37]. The potential spread and adaptation of *P. vivax* emphasizes the need to consider geographical and environmental heterogeneity when developing malaria control and intervention strategies.

While hospital-based cases had higher parasitemia, some asymptomatic community infections were also shown with high parasitemia, indicating transmission potential. In Ethiopia, *P. vivax* in Duffy-negative individuals was observed in both febrile patients and asymptomatic carriers [38,39], and some of these infections were detected with gametocytes [40]. These findings emphasize the need for comprehensive surveillance and interventions in both hospital and community settings. The use of microscopy and RDT diagnostics, though convenient, could have vastly misidentified or missed low-density *P. vivax* infections. In our study, some individuals classified as microscopy-negative showed moderate to high parasitemia by qPCR, raising concerns about the diagnostic sensitivity of microscopy and PvLDH-based RDTs, especially in Duffy-negative individuals. Similar microscopy results have been reported in a study from Indonesia [41], where microscopy detected only 5 mixed infections of *P. falciparum* and *P. vivax*, while nested PCR detected 346 cases across various clinical categories, including asymptomatic individuals and those recently treated with antimalarials. Notably, all microscopy results were negative in individuals’ post-treatment, while PCR still detected ongoing parasitemia in most cases [41]. Additionally, our findings also suggest that PvLDH-based RDTs may contribute to false-negative results, even in cases of high parasitemia. This could be attributed to defective or degraded test kits, lot variability, or antigenic variation that affects the detection capability of PvLDH in majority Duffy-negative Africa. Molecular tools like qPCR offer superior sensitivity and specificity, particularly in detecting low-density or mixed *P. vivax* infections in Duffy-negative individuals, ensuring both symptomatic and asymptomatic carriers are identified and monitored effectively.

The *PvDBP1* gene encodes a critical ligand that facilitates *P. vivax* invasion of human erythrocytes by binding to the Duffy antigen receptor, making it a key molecular marker for both species confirmation and evolutionary analysis. While previous studies in Cameroon have documented the presence of *P. vivax*, this is the first study to utilize *PvDBP1* not only to validate *P. vivax* infections in Duffy-negative individuals, but also to examine genetic variations and phylogenetic relationships of the Cameroonian isolates with global *P. vivax* populations. In the Cameroonian isolates, mutations I379L and E225K occurred at notably high frequencies. Several identified mutations, including K326E, G339D, N372K, and I379L overlapped with *Pv* populations from Southeast Asia [26]. Mutations I379L and G339D occurred at substantially higher frequencies in our dataset than other geographical isolates, suggesting regional adaptation or selective pressures in Central African parasite populations. These findings highlight the need for future functional assays to clarify their potential role in parasite invasion and virulence.

Previous studies indicated striking similarity of the *P. vivax* genome between Asian and East African parasite populations with high identity-by-descent [42]. Our prior research showed no clear genetic distinction between *P. vivaxv* in Duffy-negative and Duffy-positive hosts, indicating shared ancestry and gene flow [25,43]. This study further shows that Cameroonian and Botswanan *P. vivax* form a distinct cluster, separate from East African isolates, likely due to ecological adaptation and vector diversity. For instance, *P. vivax* parasites show strong compatibility with local mosquito vectors [44]. In Cameroon and Benin, *Anopheles coluzzii* was identified as a competent vector for *P. vivax* [45], suggesting its potential significance in sustaining transmission in West/Central Africa [46]. By contrast, in East Africa, *An. arabiensis* and *An. funestus* are primary vectors for malaria transmission. While *An. arabiensis* is more commonly associated with *P. falciparum*, there is evidence suggesting its potential role in transmitting *P. vivax* [47–49]. This vector competence supports the ecological feasibility of *P. vivax* transmission in these regions, even though the phylogenetic clustering likely reflects broader processes such as host population structure, migration history, and region-specific selective pressures rather than vector diversity alone.

Apart from vector-parasite co-evolution, host genetic factors may also shape parasite population structure. Evidence of severe population bottlenecks followed by rapid expansion in human *P. vivax*, along with gene pseudogenization (e.g., RBP2d, RBP3) supports host-specific adaptation in *P. vivax* [50]. East Africa, with a larger Duffy-positive population, likely harbors an older, more diverse *P. vivax* gene pool; whereas Central/Southern Africa may represent a portion of *P. vivax* parasites recently introduced from East Africa or constrained populations shaped by bottlenecks and the predominance of Duffy-negative hosts [29,50]. The observed genetic relatedness between Cameroonian and Botswanan isolates suggests possible recent gene flow. The Bantu expansion is the most important and well-studied migration event between Central and Southern Africa, where Bantu-speaking people migrated from West-Central Africa, eventually settling in much of the southern half of the continent and interacting with indigenous groups like the Khoisan, around 3,000–5,000 years ago [51,52]. This migration event could have shaped the parasite population structure. Beyond host genetic structure and migration history, differences in antimalarial drug pressure may also contribute to the observed clustering of Cameroonian and Botswana *P. vivax* away from East African lineages. Central African countries such as Cameroon, and to some extent Botswana, experience intense *P. falciparum* transmission and have deployed a suite of *P. falciparum*-focused interventions, including seasonal malaria chemoprevention (SMC) [53], post-discharge malaria chemoprevention (PMC) [54], intermittent preventive treatment in pregnancy (IPTp), and widespread artemisinin-based combination therapy. In such settings, *P. vivax* infections are likely to be repeatedly and passively exposed to antimalarial drugs administered for clinical *P. falciparum* episodes, including in mixed infections. By contrast, many East African contexts included in our comparison (e.g., parts of Ethiopia and Sudan) have different transmission profiles and intervention portfolios, potentially resulting in lower cumulative drug pressure on *P. vivax* [55].

It is important to note that the *PvDBP1* region II clustering of Cameroonian isolates with Botswana samples contrasts with recent findings from the Democratic Republic of Congo, where *P. vivax* sequences from the DRC were most similar to isolates from Uganda [34]. This difference highlights the heterogeneous nature of *P. vivax* emergence in Central Africa, suggesting that vivax populations in this region do not form a single homogeneous lineage but instead reflect distinct epidemiological histories and patterns of gene flow. Botswana, although reporting far fewer cases, has documented molecularly confirmed *P. vivax* infections [30], indicating that vivax parasitemia does occur even in populations that are overwhelmingly Duffy-negative. Taken together, these comparisons emphasize that *P. vivax* population structure across Central, East, and Southern Africa is shaped by localized ecological, demographic, and possibly drug-selection environments rather than by a single continental pattern.

This study has a few limitations. First, samples and related metadata were collected at a single timepoint and thus restricts our ability to assess *P. vivax* transmission over time. Second, the genetic analysis based on *PvDBP1* alone does not reflect evolutionary changes at the genome level for *P. vivax* in Duffy-negative individuals. The geographic representation of African *P. vivax* isolates, particularly samples from West Africa, is limited in the phylogenetic analysis. The apparent genetic distinctiveness of Central African isolates may change with broader sampling across the continent. Third, while our analysis assumes independence of observations, the hospital- and household-based sampling may introduce some degree of natural clustering. This may result in slightly underestimated standard errors, though it is unlikely to substantially alter the conclusions of this study.

To conclude, the detection of several *P. vivax* cases in asymptomatic individuals underscores its potential for silent transmission, emphasizing the need for enhanced surveillance in both community and hospital settings. PvLDH-based RDTs may contribute to false-negative diagnostic results, even in cases of high parasitemia. Molecular tools like qPCR offer superior sensitivity and specificity, particularly in detecting low-density or mixed *P. vivax* in Duffy-negative individuals. Our present findings call for tailored malaria control strategies that consider both geographical/environmental heterogeneity and the unique features of *P. vivax* in Duffy-negative populations.

## Supporting information

S1 Fig Parasitemia across age groups. This figure compares parasitemia levels across different age groups (<5, 5–15, > 15 years) with no significant differences observed between the age groups (ns).
(TIFF)

S2 Fig Parasitemia across sex. This figure examines the parasitemia levels between female and male participants, with no significant differences observed (ns).
(TIFF)

S1 Table Primer sequences. *Plasmodium falciparum* and *Plasmodium vivax* primers sequences.
(PDF)

S2 Table PvDBP1 sequences. Global *Plasmodium vivax* Duffy Binding Protein 1 Region II (*PvDBP1* region II) sequences used for phylogenetic comparison.
(DOCX)

S3 Table Prevalence of Mon*o-Plasmodium vivax (Mono-Pv), Mono-Plasmodium falciparum (Mono-Pf), and mixed infections (Pv + Pf*) across different sex and age groups in Bamenda, Bertoua and Buea. Hospital-based data are presented as positivity rates (%, n), defined as the proportion of individuals testing positive among those evaluated at health facilities. Community-based data are presented as prevalence rates (%, n), defined as the proportion of infected individuals within the sampled population. Percentages are calculated within each site, sex, and age stratum using the total number (N) in that stratum as the denominator. Subtotals reflect aggregated counts within each site. “NA” indicates strata with no available observations or insufficient sample size for estimation. All estimates are descriptive and derived from univariate analyses; no adjustments were made for potential confounding factors. Values in parentheses represent the number of positive cases within each stratum.
(DOCX)

S4 Table Exploratory distribution of reported symptoms among participants with *Plasmodium vivax*, *Plasmodium falciparum*, and mixed infections. This table summarizes the number of participants reporting each symptom and the corresponding distribution of total infections, mono-*Pv*, mono-*Pf*, and mixed (*Pv* + *Pf*) infections within each symptom category. Values represent raw counts. Due to small sample sizes within several symptom strata, these results are descriptive and exploratory in nature and should not be interpreted as evidence of causal or independent associations. Some symptom categories contained zero observations. Headache data were incomplete for a subset of participants, as indicated. *(data missing): 11 samples were excluded due to incomplete symptom data.
(DOCX)
