# Supplementary material for: Epidemiological insights and genetic diversity of the Duffy binding protein of Plasmodium vivax in Duffy-negative Cameroonians
Source: PLoS Negl Trop Dis. 2026 Jun 4;20(6):e0014404. doi: 10.1371/journal.pntd.0014404 (PMC13235936; doi:10.1371/journal.pntd.0014404)
Supplement: S1 Table — Plasmodium falciparum and Plasmodium vivax primers sequences. (PDF) [file pntd.0014404.s003.pdf]

Plasmodium Falciparum Primers	
Forward	TAT TGC TTT TGA GAG GTT TTG TTA CTT TG
Reverse	ACC TCT GAC ATC TGA ATA CGA ATG C
***Plasmodium vivax* Primers**	
Forward	GCT TTG TAA TTG GAA TGA TGG GAA T
Reverse	ATG CGC ACA AAG TCG ATA CGA AG
