# Supplementary material for: Epidemiological insights and genetic diversity of the Duffy binding protein of Plasmodium vivax in Duffy-negative Cameroonians
Source: PLoS Negl Trop Dis. 2026 Jun 4;20(6):e0014404. doi: 10.1371/journal.pntd.0014404 (PMC13235936; doi:10.1371/journal.pntd.0014404)
Supplement: S2 Table — Global Plasmodium vivax Duffy Binding Protein 1 Region II (PvDBP1 region II) sequences used for phylogenetic comparison. (DOCX) [file pntd.0014404.s004.docx]

| **Region / Country** | **Study reference** | **Sequencing Technology** | **GenBank accession range / IDs** |
| --- | --- | --- | --- |
| **Cameroon (Central Africa)** | ***This study*** | Sanger | PX699213 -  PX699280 |
| **Brazil (South America)** | Genetic variability and natural selection at the ligand domain of  the Duffy binding protein in Brazilian Plasmodium vivax populations | Sanger | EU812950.1 |
| **Sudan (Northeast Africa)** | ***This study*** | NGS | PX840725 –  PX840901 |
| **Ethiopia (East Africa)** | Contrasting epidemiology and genetic variation of Plasmodium vivax infecting Duffy-negative individuals across Africa | Sanger | MZ062224 -  MZ062409 |
| **Botswana (Southern Africa)** | Contrasting epidemiology and genetic variation of Plasmodium vivax infecting Duffy-negative individuals across Africa | Sanger | MZ062224 -  MZ062409 |
| **Uganda (East Africa)** | Genetic polymorphism of Duffy binding protein domain II from Plasmodium vivax in Uganda | Sanger | KX009557.1 |
| **Thailand (Southeast Asia)** | Polymorphism patterns in Duffy-binding protein among Thai  Plasmodium vivax isolates | Sanger | EF368162.1 |
| **China (East Asia)** | Genetic diversity and natural selection of Plasmodium vivax Duffy binding protein-II from China-Myanmar Border of Yunnan Province, China | Sanger | MZ765947 -  MZ766070 |

**Table S2.** **Global *Plasmodium vivax Duffy Binding Protein 1 Region II (PvDBP1* region II*)* sequences used for phylogenetic comparison.**

The Cameroon sequences represent newly generated *PvDBP1* region II amplicons from this study (Duffy-negative individuals, Bamenda–Buea–Bertoua). All other accessions were retrieved from GenBank for comparative phylogenetic analysis. The Botswana and Ethiopia accessions share the same sequence range because both datasets were released under the same GenBank submission.
