# Supplementary material for: Epidemiological insights and genetic diversity of the Duffy binding protein of Plasmodium vivax in Duffy-negative Cameroonians
Source: PLoS Negl Trop Dis. 2026 Jun 4;20(6):e0014404. doi: 10.1371/journal.pntd.0014404 (PMC13235936; doi:10.1371/journal.pntd.0014404)
Supplement: S3 Table — Hospital-based data are presented as positivity rates (%, n), defined as the proportion of individuals testing positive among those evaluated at health facilities. Community-based data are presented as prevalence rates (%, n), defined as the proportion of infected individuals within the sampled population. Percentages are calculated within each site, sex, and age stratum using the total number (N) in that stratum as the denominator. Subtotals reflect aggregated counts within each site. “NA” indicates strata with no available observations or insufficient sample size for estimation. All estimates are descriptive and derived from univariate analyses; no adjustments were made for potential confounding factors. Values in parentheses represent the number of positive cases within each stratum. (DOCX) [file pntd.0014404.s005.docx]

**Table S3. Prevalence of Mono-*Plasmodium vivax* (Mono-*Pv*), Mono-*Plasmodium falciparum* (Mono-*Pf*), and mixed infections (Pv + Pf) across different sex and age groups in Bamenda, Bertoua and Buea**.

| **Site** | **Sex** | **Age** | **Total (N)** | **Mono-*Pv*** | **Mono-*Pf*** | **Pv + Pf** |
| --- | --- | --- | --- | --- | --- | --- |
| **Hospital** |  |  |  | **Positivity rate (%, n)** | | |
| Bamenda | Female | <5 | 97 | 7.2 (7) | 22.7 (22) | 19.6 (19) |
|  |  | 5-15 | 12 | 16.7 (2) | 41.7 (5) | NA |
|  |  | >15 | 201 | 14.9 (30) | 8.0 (16) | 9.5 (19) |
|  | Male | <5 | 117 | 6.8 (8) | 14.5 (17) | 10.3 (12) |
|  |  | 5-15 | 13 | 7.7 (1) | NA | 7.7 (1) |
|  |  | >15 | 58 | 10.3 (6) | 10.3 (6) | 5.2 (3) |
|  | Sub-total |  | 498 | 10.8 (54) | 13.3 (66) | 10.8 (54) |
| Buea | Female | <5 | 29 | 6.9 (2) | 55.2 (16) | 10.3 (3) |
|  |  | 5-15 | 24 | 4.2 (1) | 33.3 (8) | 29.2 (7) |
|  |  | >15 | 130 | 12.3 (16) | 40.0 (52) | 14.6 (19) |
|  | Male | <5 | 32 | 9.4 (3) | 50.0 (16) | 15.6 (5) |
|  |  | 5-15 | 26 | 7.7 (2) | 46.2 (12) | 23.1 (6) |
|  |  | >15 | 54 | 14.8 (8) | 40.7 (22) | 22.2 (12) |
|  | Sub-total |  | 295 | 17.7 (32) | 42.7 (126) | 17.6 (52) |
| Total (%) |  |  | 793 | 10.8 (86) | 24.2 (192) | 13.4 (106) |
| **Community** | |  |  | **Prevalence rate (%, n)** | | |
| Bertoua | Female | <5 | 57 | 1.8 (1) | 42.1 (24) | 3.5 (2) |
|  |  | 5-15 | 100 | 4.0 (4) | 53.0 (53) | 6.0 (6) |
|  |  | >15 | 100 | 3.0 (3) | 35.0 (35) | 9.0 (9) |
|  | Male | <5 | 63 | 3.2 (2) | 52.4 (33) | 6.3 (4) |
|  |  | 5-15 | 107 | 5.6 (6) | 43.0 (46) | 7.5 (8) |
|  |  | >15 | 40 | 2.5 (1) | 40.0 (16) | 7.5 (3) |
| Buea | Female | <5 | NA | NA | NA | NA |
|  |  | 5-15 | NA | NA | NA | NA |
|  |  | >15 | 68 | 10.3 (7) | 14.7 (10) | 10.3 (7) |
|  | Male | <5 | 1 | 100 (1) | NA | NA |
|  |  | 5-15 | NA | NA | NA | NA |
|  |  | >15 | 44 | 15.9 (7) | 27.3 (12) | 6.8 (3) |
| **Total (%)** |  |  | 580 | 5.5 (32) | 39.5 (229) | 7.2 (42) |

Hospital-based data are presented as positivity rates (%, n), defined as the proportion of individuals testing positive among those evaluated at health facilities. Community-based data are presented as prevalence rates (%, n), defined as the proportion of infected individuals within the sampled population. Percentages are calculated within each site, sex, and age stratum using the total number (N) in that stratum as the denominator. Subtotals reflect aggregated counts within each site. “NA” indicates strata with no available observations or insufficient sample size for estimation.

All estimates are descriptive and derived from univariate analyses; no adjustments were made for potential confounding factors. Values in parentheses represent the number of positive cases within each stratum.
