# Supplementary material for: Epidemiological insights and genetic diversity of the Duffy binding protein of Plasmodium vivax in Duffy-negative Cameroonians
Source: PLoS Negl Trop Dis. 2026 Jun 4;20(6):e0014404. doi: 10.1371/journal.pntd.0014404 (PMC13235936; doi:10.1371/journal.pntd.0014404)
Supplement: S4 Table — This table summarizes the number of participants reporting each symptom and the corresponding distribution of total infections, mono-Pv, mono-Pf, and mixed (Pv + Pf) infections within each symptom category. Values represent raw counts. Due to small sample sizes within several symptom strata, these results are descriptive and exploratory in nature and should not be interpreted as evidence of causal or independent associations. Some symptom categories contained zero observations. Headache data were incomplete for a subset of participants, as indicated. *(data missing): 11 samples were excluded due to incomplete symptom data. (DOCX) [file pntd.0014404.s006.docx]

**Table S4. Exploratory distribution of reported symptoms among participants with *Plasmodium vivax*, *Plasmodium falciparum*, and mixed infections.**

| **Variables** | **Number of Samples** | **Total Infections** | ***P.***  ***vivax*** | ***P.***  ***falciparum*** | **Mixed Infections** |
| --- | --- | --- | --- | --- | --- |
| Fever | 4 | 2 | 1 | 1 | 0 |
| Chills | 0 | 0 | 0 | 0 | 0 |
| Abdominal Pain | 5 | 2 | 0 | 0 | 2 |
| Headache*(data missing) | 11 | 5 | 1 | 1 | 3 |
| Nausea | 1 | 0 | 0 | 0 | 0 |
| Diarrhea | 2 | 0 | 0 | 0 | 0 |
| Difficulty Breathing | 0 | 0 | 0 | 0 | 0 |
| Muscle Pain | 5 | 3 | 0 | 1 | 2 |
| Fatigue | 9 | 3 | 2 | 0 | 1 |

This table summarizes the number of participants reporting each symptom and the corresponding distribution of total infections, mono-*Pv*, mono-*Pf*, and mixed (*Pv* + *Pf*) infections within each symptom category. Values represent raw counts. Due to small sample sizes within several symptom strata, these results are descriptive and exploratory in nature and should not be interpreted as evidence of causal or independent associations. Some symptom categories contained zero observations. Headache data were incomplete for a subset of participants, as indicated. *(data missing): 11 samples were excluded due to incomplete symptom data.
